# Field evidence for litter and self‐DNA inhibitory effects on *Alnus glutinosa* roots

**DOI:** 10.1111/nph.18391

**Published:** 2022-08-10

**Authors:** Giuliano Bonanomi, Maurizio Zotti, Mohamed Idbella, Pasquale Termolino, Veronica De Micco, Stefano Mazzoleni

**Affiliations:** ^1^ Department of Agricultural Sciences University of Naples Federico II via Università 100 80055 Portici (Naples) Italy; ^2^ Task Force on Microbiome Studies University of Naples Federico II 80100 Naples Italy; ^3^ CNR‐IBBR institute of Bioscience and BioResources Via Università 133 80055 Portici (Naples) Italy

**Keywords:** ^13^C‐CPMAS NMR, aquatic root, C : N ratio, decomposition, epifluorescence microscopy, plant–soil feedback, root anatomy

## Abstract

- Litter decomposition releases nutrients beneficial to plants but also induces phytotoxicity. Phytotoxicity can result from either labile allelopathic compounds or species specific and caused by conspecific DNA. Aquatic plants in flowing water generally do not suffer phytotoxicity because litter is regularly removed. In stagnant water or in litter packs an impact on root functionality can occur. So far, studies on water plant roots have been carried out in laboratory and never in field conditions.
- The effect of conspecific vs heterospecific litter and purified DNA were assessed on aquatic roots of the riparian woody species *Alnus glutinosa* L. using a novel method, using closed and open plastic tubes fixed to single roots in the field with closed tubes analogous to stagnant water. Four fresh and four decomposed litter types were used and analysed on extractable C, cellulose, lignin, N content and using ^13^C‐CPMAS NMR spectroscopy.
- Inhibitory effects were observed with fresh litter in closed systems, with a positive correlation with extractable C and negative with lignin and lignin : N ratio. *Alnus* self‐DNA, but not heterologous one, caused acute toxic effects in the closed system.
- Our results demonstrate the first field‐based evidence for self‐DNA inhibition as causal factor of negative feedback between plants and substrate.

## Introduction

Leaf litter represents a substantial fraction of the plant debris periodically returning to the soil, stream, and lake systems and undergoing decomposition (Aerts, 1997). Plant litter is the main input of organic carbon and nutrients that feed the biogeochemical cycles, but leaf litter also plays an important role in shaping plant community structure (Facelli & Pickett, 1991). The presence of litter in grassland, forest and riparian vegetation has major effects as either promotion or inhibition of seed germination, seedling establishment (Jensen & Gutekunst, 2003), plant root growth and nutrition (Cuevas & Medina, 1988; Conn & Dighton, 2000), protection from desiccation (Loydi *et al*., 2013), release of mineral nutrients during decomposition to support plant nutrition (Hobbie, 2015), and the support of a beneficial microbiota in the soil systems (Hättenschwiler *et al*., 2005). Conversely, inhibitory effects of leaf litter have been reported in some studies (Xiong & Nilsson, 1999) related to a physical obstacle action (Scarpa & Valio, 2008) or, more commonly, to a chemical interference (Rice, 2012). In the latter case, plant growth inhibition may be due to a combination of nutrient starvation and chemical toxicity due to allelopathic compounds.

Existing studies agree that plant nutrient starvation mainly involves nitrogen (N) and it is caused by microbial competition when the C : N ratio of decomposing organic matter is above the threshold values of *c*. 30 (Hodge *et al*., 2000). In fact, when the leaf litter has a low N content microbes outcompete plants for mineral N uptake. The rapid microbial uptake and the incorporation of N in the microbial biomass may cause a temporary deprivation of mineral N forms in the soil (Lummer *et al*., 2012), therefore impairing plant growth. The intensity and duration of this N immobilisation depends on the litter amount and its C : N ratio and lasts from few weeks as for leaf litter, to several years when large amounts of wood is involved, for example after large disturbance by hurricanes (Zimmerman *et al*., 1995).

Direct litter phytotoxicity is also widespread, with different studies having reported an inhibitory effect for 21 (Lopez‐Iglesias *et al*., 2014), 64 (Bonanomi *et al*., 2011) and 65 (Meiners, 2014) litter types in both temperate and Mediterranean ecosystems. A wide array of allelopathic compounds has been isolated and identified, especially in water leachate of fresh litter and during the early phases of decomposition (Rice, 2012). The most common phytotoxic compounds in litter include short‐chain organic acids such as propionic and butyric acids (Armstrong & Armstrong, 1999), tannins (Kraus *et al*., 2003) and low molecular weight phenols (Li *et al*., 2010). In this regard, previous studies also clarified that litter phytotoxicity is largely affected by plant functional type and by the stage of decomposition (Bonanomi *et al*., 2017). Overall, leaf litter is usually more phytotoxic than root debris and, among plant functional types, tissues of nitrogen‐fixing species are, on average, more inhibitory than forbs, woody species, and grasses (Bonanomi *et al*., 2017). Moreover, it is also well established that the rapid degradation of the labile allelochemicals compounds and their transformation into nontoxic molecules cause a fast disappearance within weeks or few months of the litter phytotoxic effects (Chou & Patrick, 1976; Bonanomi *et al*., 2006; Dorrepaal, 2007).

Plant litter has been shown to have species‐specific autotoxic effects (Singh *et al*., 1999), and the inhibition is long lasting, up to several years, as in the so‐called soil ‘sickness’ or ‘soil fatigue’ in agriculture after repeated monospecific crops (Cesarano *et al*., 2017). Moreover, autotoxic compounds could promote the virulence of soilborne pathogens by weakening plant defences and reducing the resistance to pathogen attack, therefore creating a negative density‐dependent effect (Packer & Clay, 2000; Xia *et al*., 2015). Recently, species‐specific and long‐lasting autotoxicity has been associated with the release and accumulation of extracellular DNA (exDNA) in the litter layer and underlying soil during decomposition (Mazzoleni *et al*., 2015a). Noteworthily, a series of laboratory experiments highlighted that exDNA toxicity depends on fragment size, with the major inhibition observed for fragments ranging between 50 and 2000 bp (Mazzoleni *et al*., 2015a), that is a smear of sizes comparable with those observed after genomic DNA degradation due to decomposition process of litter materials. A recent study in the model plant *Arabidopsis thaliana* reported evidence of extracellular nonself‐DNA entering root tissues and cells down to the nuclei, with the corresponding activation of different metabolic pathways for its active processing. By contrast, no uptake of self‐DNA was observed, with the molecules remaining outside and inducing limited cell permeability, reduced chloroplast functioning, ROS production and eventually causing cell cycle arrest. Moreover, after some days, root apex necrosis and leaf chlorosis occurred only in the plants exposed to self‐DNA (Chiusano *et al*., 2021). Also, a very recent paper by Palomba *et al*. (2022) reported for the first time the occurrence of the inhibitory effects of extracellular self‐DNA on two photosynthetic aquatic microalgae: the freshwater *Chlamydomonas reinhardtii* and the marine *Nannochloropsis gaditana*. Moreover, in the former species, significant phenotypic effects were observed with formation of heteromorphic cell aggregates only in the presence of self‐DNA in the environment and never after exposure to nonself‐DNA. However, all published studies to date have been carried out under laboratory conditions (Mazzoleni *et al*., 2015b; Barbero *et al*., 2016; Duran‐Flores & Heil, 2018), with no evidence to date of exDNA toxicity under field conditions.

The impact of leaf litter on plant community structure depends on the amount, spatial distribution and chemical quality of plant debris. In other words, the expected impact of litter phytotoxicity and autotoxicity may depend on their physical distribution, cycling pathways, and decay rates in different ecosystems. For instance, in fire prone plant communities leaf litter is periodically removed and transformed into highly heterogeneous mixtures of charred materials and mineral ash (González‐Pérez *et al*., 2004). In riparian ecosystems, instead, leaf litter is continuously removed by water fluxes with spatial re‐arrangement of floating debris that create a mosaic of free areas and zones of accumulation, that is leaf litter packs (Kominoski *et al*., 2007; Peralta‐Maraver *et al*., 2011). In ecosystems with regular water flush (e.g. wetlands and marshes, floating vegetation, riparian forests and mangrove stands, seaweed and seagrasses as well as kelp forests), the litter dispersal combined with the dilution of the dissolved organic fractions leached from the same decomposing debris may drastically reduce the potential impact of litter on root growth (Mazzoleni *et al*., 2010). By contrast, a strong impact of leaf litter could be expected in proximity to local debris accumulation as occurring in leaf litter packs along rivers. In Mediterranean streams the formation of leaf litter and wood debris packs is seasonal, with litter concentration in water progressively increasing from spring to summer because of either enhanced litterfall or reduction of water discharge in July and August (Bernal *et al*., 2003).

Plant litter contributes to plant–soil feedback (Veen *et al*., 2019) by controlling nutrient cycling and by the release of allelopathic compounds, for example short chains fatty acids, alkaloids, flavonoids, phenols, tannins, terpenes, etc., as well extracellular DNA as recently demonstrated (Mazzoleni *et al*., 2015a). Various chemicals are released during decomposition and their fate is determined by abiotic factors and the activity of associated microbiota (Bonanomi *et al*., 2021). Here, we used a down‐scaling approach in which the active roots have been first exposed to the entire litter of four coexisting species encompassing a wide range of chemical traits. Then, we investigated the effect of purified self‐DNA and nonself‐DNA as putative chemicals contributing to the litter inhibition of root proliferation. To this aim, we conceived and developed a novel method to test the effect of plant litter, as well as of pure chemical compounds, on roots of tree species in field conditions in riparian ecosystems. Most previous studies have been carried out in laboratory conditions using very sensitive, short‐lived species (Macías *et al*., 2000; Weston, 2000), leaving doubts about the ecological importance of this allelopathic compounds under field conditions. Here, a new method was designed based on the use of plastic tubes fixed to single roots and filled with either leaf litter or pure chemical compounds. These devices were applied on *Alnus glutinosa* L. in a riparian system. This species was selected because it is common along rivers in the Mediterranean region and develops aquatic roots characterised by a robust structure and physical resistance, very suitable for a manipulative experiment.

In this study, two experiments were carried out to investigate the occurrence of phytotoxicity and autotoxicity on aquatic roots of *Alnus* trees. The first test addressed the inhibitory effects of litter materials either fresh or decomposed, whereas the second addressed the possibility that the responsible chemical compound was DNA released by the decaying litter. We first tested the effect of different litter types, both freshly fallen and decomposed for 6 months to represent the variability present into leaf litter pack, on root development and damage by assessing morpho‐anatomical traits. Litter chemistry was characterised by ^13^C‐cross‐polarisation magic angle spinning nuclear magnetic resonance (^13^C‐CPMAS NMR) spectroscopy (Kögel‐Knabner, 2002) and also analysed for proximate cellulose, lignin and C and N content. Moreover, the effect of water flux was explored by creating open and closed systems, allowing litter leachate to be continuously removed or maintained in contact with the tree roots analogous to stagnant water, respectively. Finally, another experiment was carried out to assess the impact of purified self‐ and heterologous DNA on *A. glutinosa* roots. Specific hypotheses tested in this study were:

1 higher inhibitory effects of fresh litter compared with decomposed materials;
2 litter inhibitory effect among species could be explained by chemical descriptors;
3 self‐DNA exerting inhibitory effect on roots of conspecific, whereas no toxicity is expected for heterologous DNA;
4 stronger litter and self‐DNA inhibitory effects in closed compared with open systems.

## Materials and Methods

### Study site description

**Fig. 1:**
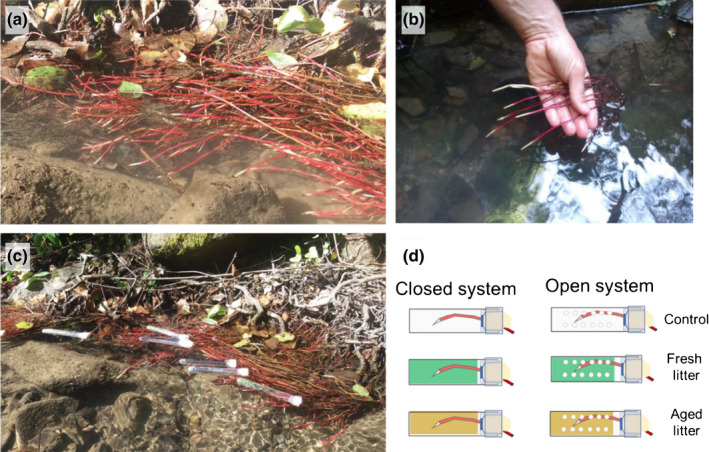
Selected images of the study site and the experimental system. (a, b) Aquatic roots of *Alnus glutinosa* in June 2018, hand indicates the scale. (c) Tubes applied to *A. glutinosa* tree to challenge roots with different leaf litter types. (d) Schematic representation of the factors included in the first experiment showing the closed (intact tubes) and open (pierced tubes with holes to allow water circulation) systems. Tubes were filled either with water (controls), fresh (green) or decomposed (brown) litter for 180 d.

The experimental activities were carried out in a stream tributary of the Alento river located in Cicerale, southern Italy (elevation of 180 m above sea level (asl), 40°19′49.17″N; 15°07′28.28″E) (Fig. 1). The riparian forest along the stream is dominated by the native trees *A. glutinosa* L., *Populus nigra* L., *Salix alba* L., *Quercus ilex* L. and the invasive, nitrogen‐fixing tree *Robinia pseudoacacia* L. The dominated vegetation layer is composed of different vines, *Hedera helix* L., *Clematis vitalba* L., *Rubus ulmifolius* Schott and *Smilax aspera* L., with a dense herbaceous layer with grasses (i.e. *Dactylis glomerata* L., *Brachypodium rupestre* Host, *Festuca drymeja* Mert.), many forbs and short‐lived nitrogen‐fixing species (details on site vegetation are reported in Incerti *et al*., 2018). Adjacent to the river, the trees alone form a narrow band, without undergrowth of other species, and typically extend their root systems into the flowing water. The study site has a Mediterranean climate with mild and wet winters and relatively hot and dry summers. Mean annual temperature is 14.8°C, with a mean monthly temperature ranging between 24.4°C (August) and 6.8°C (January). The mean annual rainfall is *c*. 1328 mm, distributed mainly in winter, autumn and spring with a notable precipitation deficit in summer, also corresponding to lower river levels.

### Species selection, decomposition experiment and chemical analyses

Litter of four species belonging to different functional groups and coexisting at the study site was selected, that is *H. helix* (evergreen vine), *F. drymeja* (perennial grass), *A. glutinosa* (deciduous, nitrogen‐fixing tree), and *P. nigra* (deciduous tree). These four litter types represent a wide range of litter chemistry in terms of nitrogen (N) and lignin and C : N and lignin : N ratios (Table 1). For trees, the litter was collected with placing nets under the canopy of selected plants at the study site (number of plants = 5, individuals randomly selected). For *H. helix* and *F. drymeja,* freshly abscised leaves were collected manually. Collected litter was transferred to the laboratory, air dried at room temperature (ventilated chamber) and thereafter stored in paper bags.

**Table 1:** Leaf litter chemical traits of the four tested plant species at two decomposition ages (0 and 180 d) assessed by elemental, proximate analyses and solid‐state cross‐polarisation magic angle spinning carbon‐13 nuclear magnetic resonance (^13^C CPMS NMR).

	*Alnus*		*Festuca*		*Hedera*		*Populus*	
	0 d	180 d	0 d	180 d	0 d	180 d	0 d	180 d
Elemental and proximate parameters								
Extractable C (%)	64.30	46.64	53.01	49.12	70.46	60.52	61.46	47.8
Cellulose (%)	18.71	21.43	26.96	13.52	23.6	10.8	22.49	11.5
Lignin (%)	13.12	23.0.71	16.15	28.56	5.72	26.6	12.51	31.22
N (%)	2.07	2.91	1.70	1.87	2.23	1.82	1.53	2.24
C : N	21.73	15.12	26.52	12.86	22.9	16.9	25.01	12.03
Lignin : N	4.53	6.42	9.51	15.31	2.89	14.55	8.22	13.94
^13^C‐CPMAS NMR parameters								
Carboxylic C 161–190 ppm	8.3	8.5	4.4	7	7.2	7.1	7.1	8.1
O‐substituted aromatic C 141–160 ppm	3.2	3.9	4.2	4.1	2.7	2.8	5.5	4
H‐C‐substituted aromatic C 111–140 ppm	11.9	13.9	12.3	12.2	5.9	8	11.6	11.3
di‐*O*‐alkyl C 91–110 ppm	8.1	6.7	17.5	10.1	8.9	6.1	12.7	8.2
*O*‐alkyl C 61–90 ppm	35.1	27.4	61.8	37.6	43.2	21.5	43.1	30.2
Methoxyl C 46–60 ppm	8.4	10.2	0.3	10.3	6.8	7.3	6.3	11.4
Alkyl C 0–45 ppm	24.4	28.2	0.1	21.3	25.3	49.3	16	29.7

Decomposed litters were produced through a decomposition experiment carried out under field conditions using the litterbag method (Berg & McClaugherty, 2014). Briefly, large (30 × 30 cm) terylene litterbags (mesh size 1.5 mm) were filled with 10 g of litter and placed over the soil surface under the canopy of *A. glutinosa*. The characteristic of the soil are reported in Supporting information Table S1. At the planned harvesting date (180 d since the start of the experiment), litterbags were collected, air dried in a ventilated chamber (35°C until constant weight was reached). At the end of the experiment, we had eight organic materials: four fresh (undecomposed litter not placed in litterbag) and four decomposed for 180 d, indicated thereafter as fresh and aged litter.

Fresh and aged litters were analysed for total organic carbon (C) and N using an elemental analyser NA 1500 (Carlo Erba Strumentazione, Milan, Italy). The content of acid‐detergent hydrolysable fraction (from this point forwards indicated as extractive C), cellulose, and proximate lignin were quantified with the method of Gessner (2005). Briefly, cellulose was quantified as hydrolysable fraction after treatment with sulphuric acid (weight loss due to 72% H_2_SO_4_), and lignin as the unhydrolyzable fraction and quantified after loss upon ignition of the fraction remaining after the H_2_SO_4_ treatment. Organic materials were also characterised by solid‐state cross‐polarisation magic angle spinning carbon‐13 nuclear magnetic resonance (^13^C‐CPMAS NMR). Analyses were carried out under the same experimental conditions to allow a quantitative comparison among obtained spectra. The analyses were carried out using a spectrometer Bruker AV‐300 equipped with a 4 mm wide‐bore MAS probe (details in Bonanomi *et al*., 2017). The spectral regions selected for analysis and corresponding to different C‐types were identified following Kögel‐Knabner (2002), Almendros *et al*. (2003) and Bonanomi *et al*. (2017). Briefly, the spectra were separated in the following seven regions: 0–45 ppm = alkyl C; 46–60 ppm = methoxyl and N‐alkyl C; 61–90 ppm = O‐alkyl C; 91–110 ppm = di‐O‐alkyl C; 111–140 ppm = H‐ and C‐ substituted aromatic C; 141–160 ppm O‐substituted aromatic C (phenolic and O‐aryl C); and 161–190 ppm carboxyl C.

### Experiment 1: root–litter interaction experiment

We selected *A. glutinosa* as a target species because of its capability to develop aquatic roots during the growing season, from May to November. These roots start to develop in May and after *c*. 3 wk of elongation phase reach a length of 10–14 cm, thereafter remaining vital without further elongation until November (G. Bonanomi, pers. obs.). First‐order water roots had a diameter of *c*. 2–3 mm and were sufficiently resistant to allow a manipulative experiment without inducing mechanical stress (Fig. 1a,b). *Salix alba* also produces a large number of aquatic roots, but they are physically fragile not allowing even careful manipulation. Therefore, this species could not be used in the same experiment. The other coexisting tree species along the stream (*P. nigra*, *R. pseudoacacia*, *Q. ilex*) were not producing aquatic roots at the time of the experiment, being distributed at a relatively higher distance from the river compared with both *Alnus* and *Salix*. At the study site, leaf packs are periodically formed in the stream, reaching high litter concentration in water. This happens especially in summer when the water flux is reduced, therefore exposing the water roots to direct contact with decomposing organic matter (Fig. S1).

The first bioassay, referred to as the ‘root–litter interaction’ experiment, was carried out in field condition to assess the short‐term impacts of leaf litter on the vitality of tree roots. To this purpose a new method was developed based on the use of plastic tubes filled with different litter types (Figs 1c,d, S2). Plastic tubes were 5 cm long with a diameter of 0.8 cm and volume of 4.5 ml. They were kept intact to create ‘closed’ systems, whereas ‘open’ systems were created by perforating alongside the tubes with ten 1.5‐mm diameter holes. The size and position of perforations allowed a free circulation of the stream water through the tubes but retaining the litter inside. In the experiment, a tube was applied to a single *A. glutinosa* root. The root was introduced in the tube and was fixed by hydrophobic cotton. The tube was filled with 0.1 g of dry litter and 4 ml of water, equivalent to a concentration of application of 25 g l^−1^. This experimental value of litter application was consistent with the levels of litterfall found in natural ecosystems (Abelho, 2001) and observed in previous studies carried out in laboratory conditions (e.g. Bonanomi *et al*., 2011). This set‐up was used because it mimics the real ecological conditions observed at the study site, allowing on the one hand the contact between both litter and its leachates with the roots, while, on the other hand between litter in clean water with the root in the closed and open systems, respectively.

Overall, the experimental design included eight litter types (four species, fresh and aged), closed and open systems, with one litter application level (25 g l^−1^). We used both fresh and aged litter because, in Mediterranean streams, litterfall inputs could be withheld in pack for several month, therefore generating accumulation of litter having different age (Fig. S1). Control tubes, both for closed and open systems, were filled only with water. Each treatment was replicated 10 times, then 180 roots were selected (eight litter types, two systems, 10 replicates plus 20 for the controls). Five adult *A. glutinosa* trees, rooted within 1 m from the stream, were selected with heights and a basal diameter range 8–12 m and 12–18 cm, respectively. For each tree, 36 roots were selected to apply all treatments to each tree with two replicates. The experiment was run in June 2018 and lasted 72 h to assess the acute effect of litter contact with roots, then the roots were cut and processed. The short duration of the experiment was decided because previous laboratory studies had demonstrated that fresh litter as well as self‐DNA exerted their detrimental effect in few hours, causing root apex necrosis (Bonanomi *et al*., 2006) and rapid changes in gene expression (Chiusano *et al*., 2021).

Immediately after excision, high resolution pictures of roots were taken with a Nikon D5 camera and damage visually assessed in percentage respect control. Thereafter, roots were conserved in ethanol (70%) for histological analysis. Subsamples of 5–10 mm length were dehydrated in an ethanol series (up to 95%), infiltrated and embedded in the acrylic resin JB4^®^ (Polysciences, Hirschberg an der Bergstrasse, Germany). Cross‐sections (5‐μm thick) were cut through a rotary microtome and mounted with mineral oil for fluorescence microscopy. Unstained sections were observed under an epifluorescence microscope (BX51; Olympus, Hamburg, Germany) with specific settings (mercury lamp, band‐pass filter 330–385 nm, dichromatic mirror 400 nm and above, and barrier filter 420 nm and above) for the detection of the UV‐induced fluorescence of phenolic compounds to highlight the occurrence of subepidermal suberised layers and accumulation of simple phenolics (Ruzin, 1999). Sections stained with 0.5% toluidine blue in water (Feder & O'brien, 1968) were observed under transmitted light microscopy to detect the occurrence of lacunae in the cortical parenchyma due to cell dehydration, collapse and cell wall disruption (North & Nobel, 1991). Digital images were captured and then processed using ImageJ software (ImageJ; United States National Institutes of Health, Bethesda, MD, USA, https://imagej.nih.gov/ij/) to quantify anatomical traits. More specifically, as a proxy of the damage (percent of damage, %*D*), the percentage of the cortical parenchyma occupied by lacunae over a given surface was quantified in three regions of the cortical parenchyma per section (De Micco *et al*., 2011; Amitrano *et al*., 2021). The three regions were selected as not consecutive sectors of 60° to cover the whole root surface. The thickness of epidermal and subepidermal layers (TEL) as well as the number of layers (NEL) was quantified in three regions of the root along three transects at 120° from each other. Stele and whole root diameter were measured along three radii of the root as well, and their ratios were calculated (S : R).

### Experiment 2: self‐DNA and nonself‐DNA effect on *A. glutinosa* roots

The second experiment was conducted under field conditions with the aim of assessing the short‐term impact of self‐DNA and nonself‐DNA on vitality of tree roots. To this purpose we used the same method applied for leaf litter (Fig. S2), but tubes were filled with purified DNA. The experimental design included two different DNA types, self‐DNA extracted from *A. glutinosa* leaf and nonself‐DNA extracted from *F. drymeja* leaves. Nonself‐DNA from *F. drymeja* was used because this species naturally coexists with *A. glutinosa* and it is also rather phylogenetically distant. DNA was extracted using cetyltrimethylammonium bromide (CTAB) methods. DNA was fragmented by sonication and applied in the tubes at concentration of 50 ppm. Selected DNA concentrations were within the range used by previous laboratory studies (Mazzoleni *et al*., 2015a,b). The experiment was carried out using both closed and open systems, with a single application rate (50 ppm) and 10 replicates. Control tubes, both for closed and open systems, were filled only with water. A total of 60 roots was selected (two DNA types, two systems, 10 replicates plus 20 for the controls). It should be noted that the leakage of the DNA solutions in the open system is certainly more rapid compared with the labile fraction in the litter treatments. Four adult trees of *A. glutinosa* rooted within 1 m from the stream, were selected, with same heights and basal diameter as those used in the first experiment. For each tree, 15 roots were used to apply all treatments to each tree. The experiment was run in June 2019 and, also in this case, the treatment duration was 72 h and then roots were excited and processed as previously described.

For histological analysis, 10 root fragments, 4 cm long, per treatment were immediately fixed in ethanol 70%. Semithin cross‐sections (10–15‐μm thick) of roots were cut along the whole fragment by means of a sliding microtome, mounted in mineral oil for fluorescence microscopy and analysed through epifluorescence microscopy as reported previously. In this specific case, observations were targeted to the detection of the occurrence of suberised cell walls (white‐violet autofluorescence), accumulation of simple phenolics (yellow‐orange autofluorescence) and of signs of plasmolysis (shrunken cell walls) in different tissues.

### Data analyses

Data analysis of visual and histological assessed root damages and root traits were designed to test the possibility that litter decomposition stage and type (either conspecific or heterospecific) can influence the normal development of aquatic roots of *A. glutinosa*, and to assess if the effect was modulated by water flow in the experimental system. The resulting data were analysed for significant changes in means by univariate factorial ANOVA. Considering that aleatory conditions such as physiology or age of different specimens of *Alnus* plants might affect the results, we treated *Alnus* individuals as a random factor, whereas litter type, litter age, water flow in either open or closed systems, as controlled factors in our experimental plan, were considered fixed. The interactive effects between the random (*Alnus*) and fixed (litter type, litter age, water flow) factors were estimated. Interactions between fixed factors were also assessed by factorial comparison to observe how the combined effect of experimental factors affected root damage. In support of the factorial ANOVA results, we ran automatic linear modelling (ALM) with a model selection to identify the predictor with stronger effects on root damage. ALM was performed using Spss software (IBM SPSS Statistics, Armonk, NY, USA) using the ‘LINEAR’ function with best subset criteria and selection based on lowest values of corrected Akaike information criteria (AIC_c_). We ran two separated ALM applications using root damage as the response variable in the model. The first assessed the importance of experimental factors with significant effect on data, including in the model litter type, litter age, and water flow as predictors (*Alnus* was excluded being nonsignificant in factorial ANOVA). The second run was performed on histological data (%*D*, TEL, NEL and S : R) to assess the best predictors among root anatomical traits of root damage. Previous to statistical analysis, both data of visual and histological assessed root damages and root traits were transformed in percentage with respect to the control and successively arctangent transformed to avoid heteroskedasticity biases. Factorial ANOVA was also applied on data from the self‐DNA experiment to test if the application of different DNA types affected the state of health of the aquatic roots, with *Alnus* plant as a random factor, and DNA treatment, either self‐DNA or nonself‐DNA applied to roots, as a fixed effect. Also, data from this second experiment were arctangent transformed. Results of factorial ANOVA were furtherly tested for significant variations by post hoc Duncan test (*P*‐values < 0.05) evaluating the differences in the means of the different experimental conditions, specifically litter type, litter age and either closed or open water flow for the first experiment, and DNA and water flow in the second experiment. Statistical analysis was performed by means of statistica 10 software. Heat‐plots were built based on Pearson correlation coefficients between values of visual root damage and chemical characteristics of litters. Significant correlations were assigned based on Pearson's *r* of *P* > 0.05. Also, for the second experiment we run ALM to assess the strongest predictors on the response variable (root damage) by the same approach used for the first experiment. In this case, we selected DNA treatment and water flow as significant predictors from factorial ANOVA.

## Results

### Litter chemistry

Litter chemistry varied among species and with decomposition time (Table 1). Among litter types, *F. drymeja* had the highest lignin : N ratio, whereas *P. nigra* recorded the lowest N content. Differently, the nitrogen‐fixing *A. glutinosa* and *H. helix* showed higher N content coupled with low C : N and lignin : N ratios. *A. glutinosa* had the highest concentration of extractable C, *F. drymeja* the lowest with intermediate values for *H. helix* and *P. nigra*. As expected, C : N ratio, extractable C and cellulose content significantly decreased with litter age for all species. By contrast, lignin content and lignin : N ratio generally increased with litter age, with higher rates for *H. helix*. During decomposition, N concentration increased for all species but for *H. helix*.

^13^C‐CPMAS NMR spectra highlighted consistent changes of litter chemistry during the 180 d of decomposition (Table 1). First, the relative abundance of O‐alkyl C and di‐O‐alkyl C fractions largely corresponding to sugars and polysaccharides, decreased during decomposition for all species, with greater reduction for *H. helix*. By contrast, the relative content of the alkyl C fraction, characteristic of lipid, waxes, and cutins produced by microbial turn‐over, increased for all species, with relatively higher values for *H. helix*, *F. drymeja* and *P. nigra*. The relative abundance of aromatic C showed negligeble changes, whereas the carboxylic C fraction showed a minor increase for *F. drymeja* and *P. nigra*.

### Leaf litter effect on *A. glutinosa* roots

**Fig. 2:**
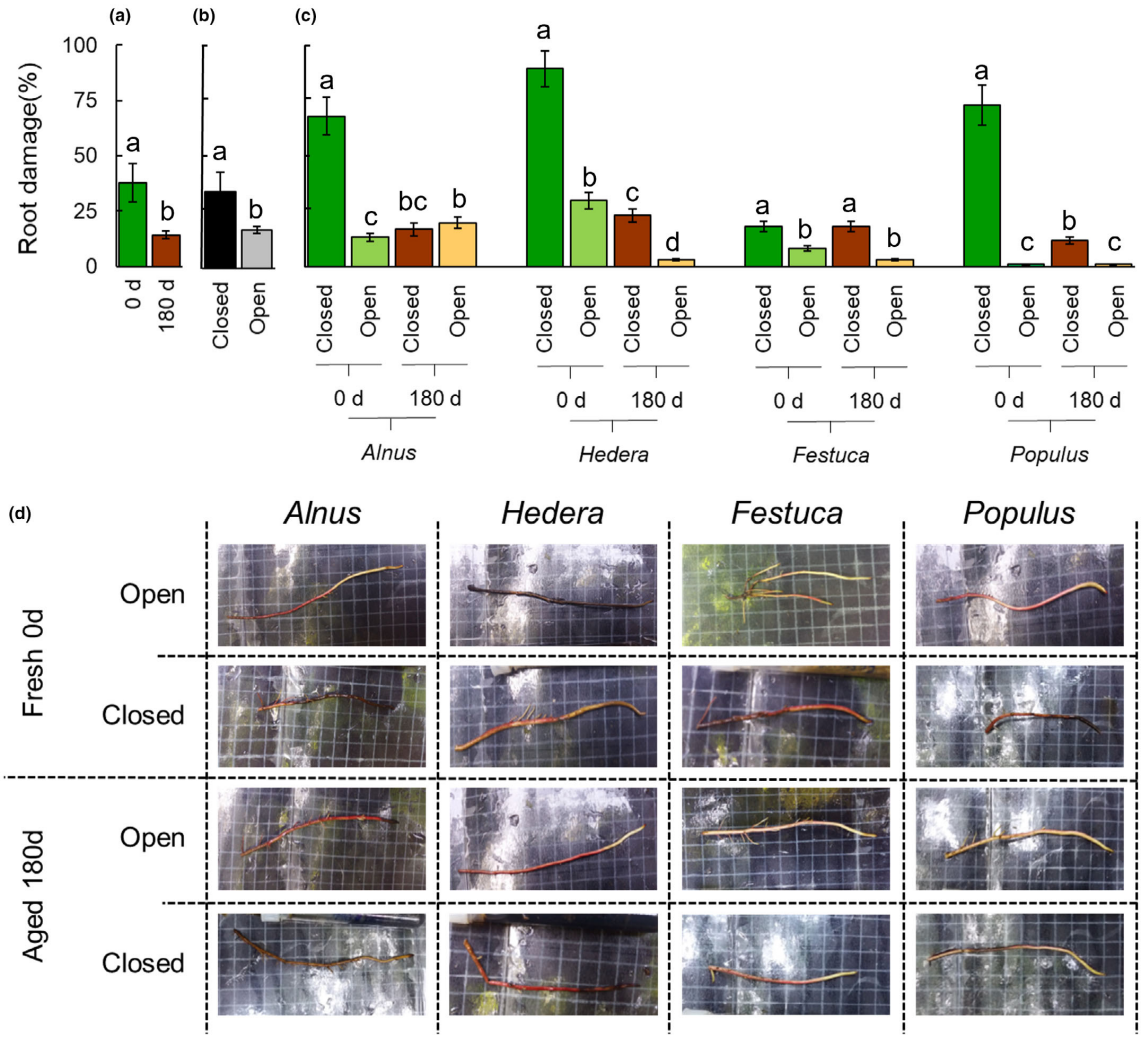
Effects of eight litter materials, either fresh (green) or after 180 d of decomposition (brown), on *Alnus glutinosa* roots in closed and open systems. (a) Root damage in fresh and decomposed litter, values are species average across. (b) Root damage in the closed and open systems, values are the average of species and decay rate. (c) Root damage for each litter type in the closed and open systems. Error bars represent ± SD. (d) Selected pictures of *A. glutinosa* roots for each treatment. Within each plot, different letters indicate statistically significant differences (Duncan test, *P* < 0.05).

The most representative predictor of the damage of litter on *A. glutinosa* roots was the type of systems that is, open vs closed (*F* = 233.291, *P* < 0.001) (Tables S2, S3), followed by litter type (*F* = 50.303, *P* < 0.001) (Tables S2, S3). Decomposition times, despite resulting significant in factorial ANOVA, were considered minor effects with respect to the other predictors in LINEAR modelling (litter age, *F* = 14.360, *P* = 0.018) (Tables S2, S3). Random factor as *Alnus* plant do not affect experimental results (*F* = 1.656, *P* = 0.774) (Table S2). Exposure to fresh plant litter produced higher macroscopic root damage compared with decomposed materials (Fig. 2a). Root lesion occurrence rates were dramatically lower in the open system compared with the closed one, for both fresh and decomposed litter (Fig. 2b). Overall, all fresh litter caused significant root damage in the closed system, but the effect varied according to litter type, being highest for *H. helix*, lowest for *F. drymeja* and intermediate for *A. glutinosa* (Fig. 2c). In the open system, instead, fresh and decomposed litter caused low root damage. Aged litter of *F. drymeja*, *H. helix*, and *P. nigra* in the open system caused very low root damage, whereas conspecific *A. glutinosa* litter caused the highest damage compared with the other litter (*P* = 0.039 vs *F. drymeja*, and *P* < 0.001 for *H. helix* and *P. nigra*; Fig. 2c; Table S4). Further details of specific comparison within different treatments are reported in Table S4.

In the closed systems, the occurrence rates of lesions in the roots of *A. glutinosa* roots were positively correlated with the extractable C and C : N ratio (Fig. S3) and negatively correlated with the lignin content and lignin : N ratio. N and cellulose concentrations did not show significant correlations with root damage in closed systems. In the closed systems, we observed weaker positive correlations between root damage and extractable C and C : N ratio, whereas a negative correlation was found with lignin content and lignin : N ratio. In the open system, no positive correlation was found, whereas the negative correlation with lignin and the lignin : N ratio was still significant. Among ^13^C‐CPMAS NMR regions, root damage was negatively correlated with aromatic C fractions both in the open and closed systems.

Histological analysis added details on the root lesion reported by the visual assessment.

All the parameters measured were significantly affected by water flow irrespective of whether they were in open or closed systems. In addition, litter type was the most effective condition (*F* = 14.9, *P* < 0.001) for percentage of damage in root sections (%*D*), whereas litter age was shown to slightly affect the number of epidermal cells (NEL) (*F* = 7.4, *P* = 0.049). The conjunction of the effect from litter age and litter types extensively affected *D*% (*F* = 24.6, *P* < 0.001), TEL (*F* = 5.177, *P* = 0.016), NEL (*F* = 7.355, *P* = 0.005), but not the S : R ratio (*F* = 2.726; *P* = 0.090) (Table 2). Comparing histological data as a predictor of root damage, ALM evidenced that the most representative predictors were the percentage of damage in root section (%*D*) followed by thickness of epidermal layer and the S : R ratio (Table S5), whereas NEL showed no contribution to the model. In detail, on the application of fresh litter, undecomposed materials determined the occurrence of higher damage in the closed system for *A. glutinosa* litter that corresponded to a significant lower TEL, due to a lower number of cell layers. No significant differences were detected in terms of percentage damage between the two systems in other litter types, with the only exception for *Populus* litter. Higher thickness and number of epidermal/subepidermal layers were also found for the closed system of *F. drymeja* litter (Table 2). For decomposed litter, higher levels of damage, were accompanied by lower values of TEL and NEL in *Alnus* in open system, whereas the opposite trends were observed in the other litter types between the different systems. No differences were observed between treatments in terms of occurrence of UV‐induced fluorescence of phenolic compounds, neither in the subepidermal suberised layers nor as accumulation of simple phenolics in the various tissues.

**Table 2:** Morphoanatomical traits of roots *Alnus glutinosa* subjected to the eight litter treatments, either fresh litter (0 d decomposition) or decomposed litter (180 d of decomposition) in open and closed systems.

	System	%*D* (%)	TEL (μm)	NEL	S : R
*Control*	Open	1.97 ± 2.95 a	375.08.41 ± 82.67 a	5.22 ± 0.97 a	0.32 ± 0.04 a
	Closed	0 ± 0 a	342.56 ± 64.12 a	4.78 ± 0.83 a	0.29 ± 0.04 a
Fresh litter (0 d)					
*Alnus*	Open	0 ± 0 a	231.55 ± 60.86 a	4.44 ± 0.53 a	0.23 ± 0.04 a
	Closed	21.31 ± 15.32 b	149.38 ± 24.12 b	3 ± 0.71 b	0.24 ± 0.03 a
*Festuca*	Open	0 ± 0 a	183.61 ± 36.96 a	3.78 ± 0.67 a	0.24 ± 0.02 a
	Closed	0 ± 0 a	269.44 ± 40.99 b	4.67 ± 0.71 b	0.26 ± 0.02 a
*Hedera*	Open	5 ± 0 a	149.18 ± 16.25 a	3.11 ± 0.33 a	0.25 ± 0.01 a
	Closed	3.64 ± 5.28 a	176.74 ± 32.11 a	3.33 ± 0.5 a	0.28 ± 0.03 a
*Populus*	Open	0 ± 0 a	216.81 ± 28.39 a	4.44 ± 0.53 a	0.25 ± 0.04 a
	Closed	5.59 ± 6.97 a	224.52 ± 43.58 a	4.33 ± 1 a	0.29 ± 0.02 a
Decomposed litter (180 d)					
*Alnus*	Open	5.46 ± 3.86 a	180.81 ± 25.44 a	3.22 ± 0.44 a	0.25 ± 0.02 a
	Closed	1.37 ± 1.68 a	247.30 ± 63.79 b	4.56 ± 0.73 b	0.26 ± 0.01 a
*Festuca*	Open	1.4 ± 2.1 a	246.79 ± 41.11 a	4.33 ± 0.71 a	0.25 ± 0.01 a
	Closed	2.55 ± 2.48 a	206 ± 47.70 a	3.56 ± 0.53 b	0.28 ± 0.03 a
*Hedera*	Open	0 ± 0 a	212.74 ± 42.72 a	4 ± 0.5 a	0.26 ± 0.01 a
	Closed	5.03 ± 7.54 a	240.66 ± 48.75 a	4.78 ± 1.09 b	0.26 ± 0.03 a
*Populus*	Open	0 ± 0 a	192.46 ± 49.03 a	3.33 ± 0.5 a	0.23 ± 0.02 a
	Closed	0 ± 0 a	243.15 ± 43.48 a	4.67 ± 0.5 b	0.26 ± 0.05 a

Values are showed as means and standard deviation. Different letters indicate significant (*P* < 0.05) differences between open and closed systems based on the Duncan test. Percent of damage (%*D*), thickness (TEL) and number (NEL) of epidermal and subepidermal layers, ratio between stele and root diameter (S : R).

### Self‐DNA and nonself‐DNA effect on *A. glutinosa* roots

**Fig. 3:**
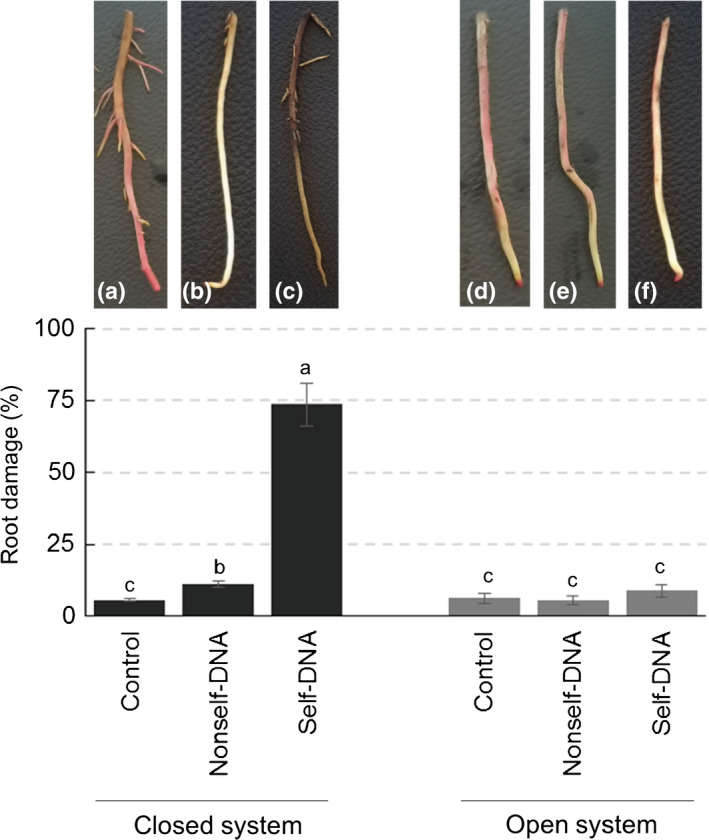
Effects of nonself‐DNA from *Festuca drymeja* and self‐DNA of *Alnus glutinosa* on roots of *A. glutinosa* after 72 h of exposure under field conditions in open and closed systems. Nonself‐DNA and self‐DNA were applied at concentration of 50 ppm. Photographs are examples of roots at the end of the experiment: (a) control in the closed system; (b) heterologous DNA in the closed system; (c) self‐DNA in the closed system; (d) control in the open system; (e) nonself‐DNA in the open system; (f) self‐DNA in the open system. Data are the mean and standard deviation; *n* = 10 for each bar; different letters indicate statistically significant differences (Duncan test, *P* < 0.05).

Analysis of experimental data showed that DNA application affected the health state of *Alnus* roots (*F* = 20.0, *P* = 0.002) (Table S6), whereas water flow had an effect only when coupled with DNA exposure. Indeed, linear modelling evidenced that DNA application best predicted root damage in the experiment (Table S7). In detail, the exposure to fragments of self‐DNA, but not of nonself‐DNA, caused substantial root damage only in closed systems and not in the open ones (Fig. 3; Tables S8, S9). In the absence of water wash‐out, the root damage level was very significantly higher in the presence of self‐DNA compared with nonself‐DNA (Table S9).

**Fig. 4:**
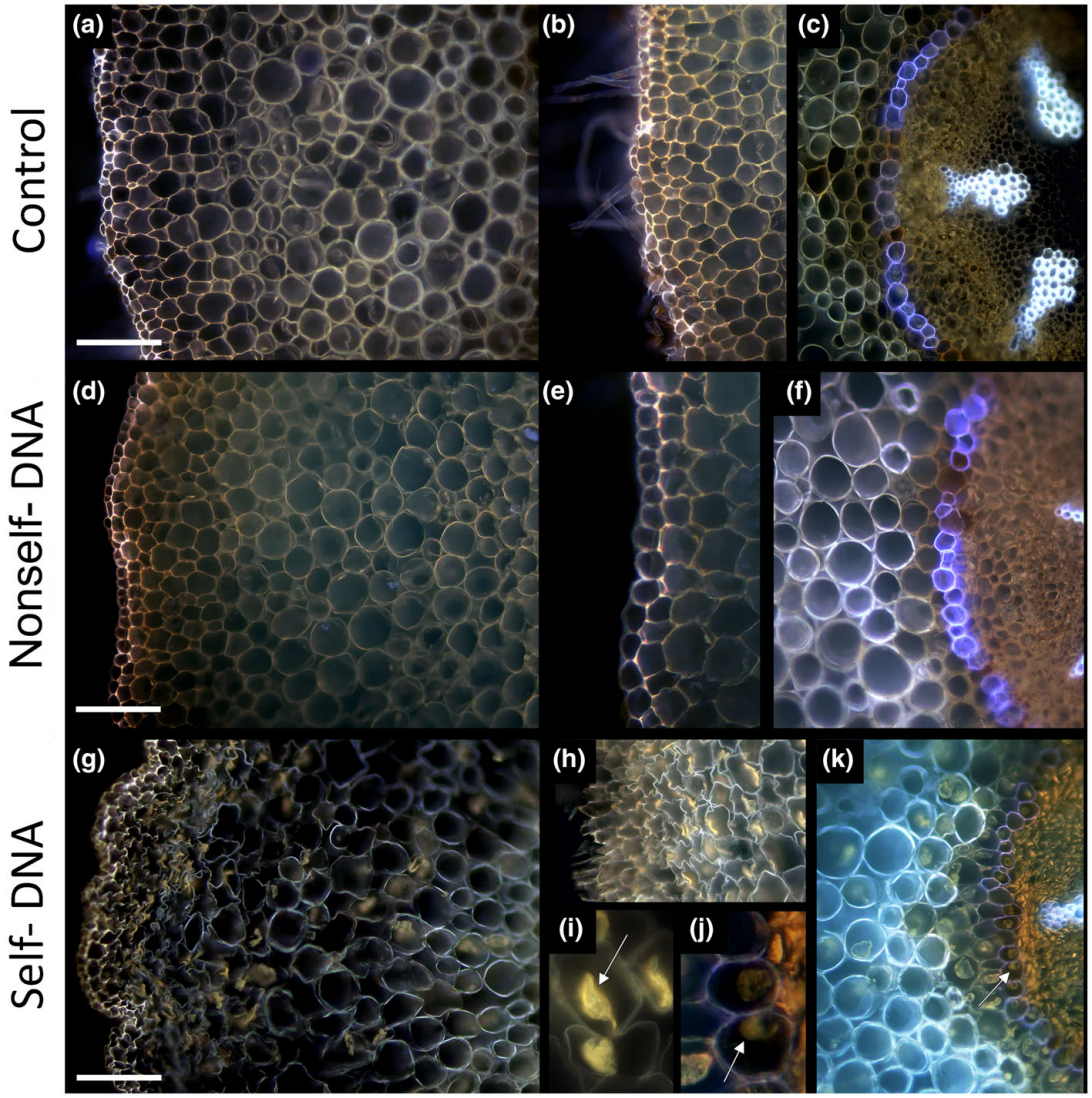
Epifluorescence microscopy views of cross‐sections of control roots (a–c), nonself‐DNA roots (d–f) and self‐DNA roots (g–m). Details of plasmolysed cells rich in simple phenolics are indicated in (h, i, j), with arrows pointing to shrunken protoplasts in (i, j, k), due to cell membrane detachment from cell walls. Note the white‐violet autofluorescence of suberin and yellow‐orange fluorescence of simple phenolics linked with cell membranes. Bar, 100 μm.

Microscopy observations showed that roots of plants in the control (Fig. 4a–c) were similar to those treated with nonself‐DNA (Fig. 4d–f). Instead, roots of plants treated with self‐DNA had an altered structure in the tissues of the cortical cylinder (Fig. 4g,m). Roots challenged with self‐DNA were characterised by very shrunken cells at the outermost cell layers (epidermis and subepidermal layers), indicating severe plasmolysis (Fig. 4g,m). This phenomenon occurred also in the parenchyma cells of the cortical cylinder up to the endodermis (Fig. 4i,l, arrows). All the roots showed autofluorescence due to suberised cell walls in the endodermis, exodermis and cortical parenchyma cells. The shrunken protoplasts in plasmolysed cells in self‐DNA roots appeared as a bright‐yellow fluorescence typical of simple phenolics linked with cell membranes.

## Discussion

### Litter effects on aquatic roots

In agreement with several previous laboratory studies (Patrick, 1971; Putnam *et al*., 1983; Rice, 2012), we found that fresh litter causes severe and acute damage to *A. glutinosa* roots after 72 h of exposure. Most previous works have recorded the inhibitory effects of fresh litter in laboratory bioassay or, in a few cases, in pot experiments with litter added to the soil (Tagliavini & Marangoni, 1992; Van der Putten *et al*., 1997; Dorrepaal, 2007). Here, we further provide evidence of the inhibitory effect of fresh litter, extending this evidence to aquatic roots of a riparian tree. As reported under *in vitro* conditions (Bonanomi *et al*., 2011) the litter inhibitory effect largely varied with plant species and decomposition time. In detail, *H. helix* was the most toxic litter, whereas *F. drymeja* the least. In our case, the higher toxic effect of *H. helix* litter compared with the other litter types was confirmed also by microscopy analyses that showed the occurrence of perturbations in anatomical traits in both open and closed systems. In the *A. glutinosa* and *P. nigra* litters, these perturbations were observed only in the closed system, whereas *F. drymeja* litter was confirmed to not trigger anatomical anomalies. Moreover, after leaves were partially decomposed, the harmful effect tended to disappear in all four tested litter materials. The present findings are fully consistent with previous studies that reported the connection during decomposition between litter chemistry and the inhibitory effects on plant growth (Bonanomi *et al*., 2011). Metabolomic studies have demonstrated the chemical complexity of leaf litter, being composed of hundreds of different molecules (Wickings *et al*., 2012; Wallenstein *et al*., 2013). During the initial phase of decomposition, the plant tissues are broken down, releasing the cell contents including many compounds such as sugars, amino acids, secondary metabolites and nucleic acids. Evidence from both proximate and ^13^C‐CPMAS NMR data have indicated that the occurrence of root damage was positively correlated with the extractable C fraction, and negatively with lignin and aromatic C compounds. Interestingly, during decomposition, all litter types became depleted of the extractable C, being used by the microbiota, and enriched in lignin‐like compounds that do not exert an inhibitory effect on plant roots (Kögel‐Knabner, 2002; Preston *et al*., 2009; Bonanomi *et al*., 2017). The importance of the labile C fraction is highlighted by the higher negative correlation found in closed compared with open systems. These results suggest that soluble, highly inhibitory compounds that are rapidly released during plant cells breakdown are effectively removed by flowing water, with a minor impact from the lignified and solid fraction of litter that remains in direct contact with the aquatic roots. Conversely, our results from closed systems demonstrated that the labile C fraction can exert its inhibitory effect when litter accumulates in stagnant water or in proximity to litter packs in the stream. In this regard, the generation of anoxic conditions in the closed system may contribute to the observed root damage. It is well known that litter decomposition under either anaerobic or poorly aerobic conditions follows different pathways and producing more phytotoxic and persistent compounds, including short‐chain organic acids and sulphides (Armstrong & Armstrong, 1999; Bonanomi *et al*., 2006; Lamers *et al*., 2013). Monitoring dissolved oxygen, redox potential and the presence of organic acids in the experimental tubes would be useful to assess the relative contribution of oxygen‐deficit with respect to the presence of either allelopathic compounds or self‐DNA in causing root damage.

Root growth inhibition by plant litter and organic amendment has been attributed to N immobilisation caused by microbes outcompeting the plant uptake capability. In detail, litter with a C : N ratio of more than 30 could temporarily reduce the N availability in soil in a time frame ranging from weeks to years (Zimmerman *et al*., 1995; Pansu & Thuriès, 2003). However, this hypothesis is not supported by our study for two reasons. First, all eight tested litter types had a C : N ratio less than the supposed threshold of 30, but still showed high levels of toxicity. Indeed, in closed systems we found a negative correlation between root damage levels and N concentration, with *F. drymeja* being the least toxic, despite its relatively higher C : N ratio. Second, we observed extensive root necrosis in a time frame (72 h) that was too short to allow a substantial N immobilisation by microbes (Hodge *et al*., 2000; Bonanomi *et al*., 2019). Overall, our findings clearly indicated that, at least in the observed experimental conditions and tested litter types, the root damage was not caused by a reduced N availability.

Most previous studies on litter allelopathic effects have been criticised because of the use of fast‐growing and sensitive target species that had little, if any, ecological relations with the studied litters. Here, the aquatic roots of *A. glutinosa* appeared to be very sensitive to litter materials of four coexisting species. Extensive root necrosis was evident over a short time span of contact with leaf litter and associated dissolved organic fraction. Assessing the relative species sensitivity to different litter type is a challenging task and few limited studies have systematically addressed this issue. A recent work analysed 36 different litter types on 14 plant species used as bioassays. This revealed a species‐specific response, with annual plants far more sensitive than long‐lived woody species (Bonanomi *et al*., 2017). An early study (van der Putten, 1997) evaluated the growth of three emergent macrophytes, *Phragmites australis*, *Typha latifolia* and *Glyceria maxima* in pots amended with *P. australis* litter. The study showed a relatively higher toxicity on conspecific litter, but also a lower sensitivity to litter inhibitory effect in submerged species.

Negative growth effects of litter or accumulated organic matter have also been reported for macrophytes such as *T. latifolia* (McNaughton, 1968), *Scirpus maritimus* (Clevering & Van der Putten, 1995) and *P. australis* (Armstrong & Armstrong, 1999). Moreover, several aquatic plants such as *Lemna minor* (Einhellig *et al*., 1985) and *Lepidium sativum* (Gehringer *et al*., 2003), among many others reviewed in Mohan & Hosetti (1999) have been routinely used for laboratory bioassays in allelopathy studies because of their sensitivity to organic chemicals. This body of evidence suggests that floating plants, macrophytes and aquatic roots of riparian tree species are very sensitive to litter phytotoxicity compared with terrestrial plant species. In general terms, this seems logical in evolutionary terms because aquatic species are not in contact with litter and do not stand high concentration of dissolved organic fractions released by decomposing litter, as do plants in soil. Further statistical evidence is needed to support the hypothesis of aquatic plants being more sensitive than terrestrial plants to litter phytotoxicity. However, it is rather obvious that a root growing in a litter‐rich forest floor has to face a much more complex chemical environment compared with the diluted and simpler conditions experienced in aquatic ecosystems.

The concept of higher litter sensitivity in aquatic plants may have relevant implications for ecosystem functioning in relation to changes in water hydrological regime. Streams, ponds, lagoons, and lakes in a Mediterranean climate are naturally subject to large fluctuations in water levels because of the seasonality of precipitation and evaporation balance (Gasith & Resh, 1999). In this context, aquatic root systems may periodically be subjected to desiccation during summer associated with the accumulation of litter and higher concentrations of dissolved organic carbon (Vazquez *et al*., 2011). Root lesions induced by the phytotoxicity of accumulated litter may produce negative impacts at the stand level. For example, the occurrences of stand die‐back of *P. australis* have been associated with litter accumulation and the consequent autotoxic effect on root systems, both in temperate (Armstrong & Armstrong, 1999) and Mediterranean climates (Gigante *et al*., 2011). Recently, stand die‐back of several plants in lagoon and saltmarsh has become more frequent and intense (Alber *et al*., 2008). In this regard, our study was limited to only one species exposed to a single litter application rate, and more work is needed to properly appreciate the impact of litter accumulation on aquatic root system functionality. Future studies are required for an effective scale‐up of the potential effects of litter phytotoxicity and autotoxicity, focusing on the interaction with fluctuation of hydrological regimes.

### DNA effects on aquatic roots

In the last decade, experimental evidence has demonstrated the inhibitory effects of self‐DNA on different organisms ranging from bacteria, algae, fungi, insect and protozoa (Mazzoleni *et al*., 2015b), up to higher plants (Mazzoleni *et al*., 2015a; Barbero *et al*., 2016). However, despite the growing evidence of self‐DNA toxicity, the underlying cellular and molecular mechanisms remain poorly understood (Chiusano *et al*., 2021). In this regard, Cartenì *et al*. (2016) proposed that the mixture of self‐DNA fragments produced during decomposition of the nuclear genome may interfere or inhibit cell functionality. The study by Chiusano *et al*. (2021) in *A. thaliana* provided some hints to the putative mechanisms of the observed effects of extracellular DNA. There is evidence that root tip cells are capable of distinguishing between self‐DNA and nonself‐DNA, with the latter entering the root tissues and cells, whereas self‐DNA remains outside and creates a cascade of events, leading to a generalised inhibitory effect, including limited cell permeability, ROS production affecting electron transport and, finally, stunted growth, fine root death and leaf discoloration. Beyond the proposed underlying mechanisms, it is evident that all available experimental evidence was obtained under highly controlled laboratory conditions. Here, for the first time, we have extended the evidence of self‐DNA toxicity to field conditions, also showing severe signs of stress at the histological level. Notably, nonself‐DNA from the phylogenetically distant *F. drymeja* caused no detrimental effects on *A. glutinosa* roots, a result consistent with the low toxicity observed for the whole grass litter. Instead, self‐DNA toxicity was responsible for a violent plasmolysis occurring throughout the epidermal layers, cortical parenchyma and endodermis, suggesting a prompt inactivation of water absorption. The lack of plasmolysis signs for nonself‐DNA suggests that the self‐DNA‐induced plasmolysis might not be linked to osmotic stress, but more likely to be linked to some specific loss of functionality of cell membranes. Indeed, the occurrence of high yellow fluorescence at the membrane level in the self‐DNA treated roots indicated the increased deposition of simple phenolics that is a common response of tissues subjected to abiotic and biotic stress (Lattanzio *et al*., 2008).

### Conclusions

In this study we conceived, developed and tested a novel method to assess the effect of leaf litter and self‐DNA on roots of riparian trees in field condition. We present evidence that leaf litter has an age‐dependent effect on *A. glutinosa* roots. A major inhibitory effect was found with fresh litter and in the closed system that mimics stagnant water. By contrast, after decomposition, litter becomes almost harmless even in the closed system lacking water recycling. The litter inhibitory effect was satisfactorily explained by chemical descriptors, with a depletion of extractable C and an enrichment of lignin and other aromatic decomposed compounds during decomposition. In other words, litter that was rich in extractable C was more toxic compared with more lignified plant tissues. In a second independent experiment, the novel method demonstrated that self‐DNA, but not heterologous DNA, caused an acute toxic effect on *A. glutinosa* roots in closed systems. Differently, in open systems, by a method that mimicked the conditions present in streams and rivers with flowing water, the harmful effects of fresh litter, as well that of self‐DNA, were dramatically reduced. We are aware that the implications of our findings, for the understanding of litter impact on plant community structure and diversity, are limited by the use of a single target species (i.e. *A. glutinosa*) and that only eight litter types used at one application rate cannot fully represent the variable conditions faced by aquatic roots in real ecosystems. However, we provided for the first time a method that allows the study of the impact of leaf litter, as well as of pure chemical compounds at the scale of primary roots under field conditions. Indeed, the functional consequence for the whole tree would depend on the number of roots involved. In our study system, it has been demonstrated that local litter accumulation is likely to impair the functionality of aquatic roots of *A. glutinosa*, especially when their role is more important for water uptake, that is, when the stream hydrological regime is reduced during the dry season. Future studies are needed to fully understand the ecological implications of litter accumulation and the associated release of the dissolved organic fraction, under fluctuating hydrological regimes, when causing vegetation stand die‐back. Moreover, our findings ask the question if, and under what circumstances, the self‐DNA inhibitory effect would be relevant in determining plant community structure and functioning. The available experimental evidence is consistent with the model proposed by Mazzoleni *et al*. (2010) that explains the formation of stable, highly productive monospecific stands only in flowing salt‐ and freshwater conditions, irrespectively of latitude, because of continuous removal of self‐DNA molecules due to their water solubility. Further research work will be necessary to investigate this subject in more species, with different phylogenetic distances and various concentration levels of the environmental DNA.

## Author contributions

GB and MZ designed and supervised the work, MZ performed the experiments and data analysis, MI supported the field work and did the litter decomposition experiment, PT did the DNA extraction, VDM performed microscopy analyses, SM coordinated the project on self‐DNA toxicity. GB, MZ and SM wrote the paper. All authors contributed to specific parts of the manuscript and approved it in the current version.

## Supporting information


**Fig. S1** Selected pictures of the study site in June 2018 showing the formation of leaf litter and wood debris packs, also note the amount of standing litter in the ponds.
**Fig. S2** Picture of the study site in June 2019 showing a typical pond in the stream with a large *Alnus glutinosa* tree (right, upper corner).
**Fig. S3** Heat‐plot of correlation (Pearson’s *r*) between *Alnus glutinosa* root damage and litter chemical descriptors in either closed or open experimental systems.
**Table S1** Physical and chemical parameters of the soil of the study site.
**Table S2** Results of univariate factorial ANOVA of variation of root damages, and histological sections depending to *Alnus* plant as random factor and litter type, litter age, water flow if in open/closed systems as fixed factors and interactive effect between the treatments.
**Table S3** Results of automatic linear modelling (ALM) with model selection on arctangent transformed root damage data as response variable and ‘water flow’ and ‘litter species’ as categorical predictor.
**Table S4** Post hoc results from Duncan test in variation of root damages in Alnus roots treated with litters of different age (days of decomposition) and type (species) and in different water flow if in open/closed systems.
**Table S5** Results of automatic linear modelling (ALM) with model selection on arctangent transformed root damage data as response variable and ‘%*D*’, ‘TEL’, ‘S : R’, and ‘NEL’ as categorical predictor from histological analysis of roots of *Alnus glutinosa*.
**Table S6** Results of univariate factorial ANOVA of variation of root damages after application of self‐DNA and nonself‐DNA, depending on the *Alnus* plant as a random factor and DNA, water flow if in open/closed systems as fixed factors and interactive effect between the treatments.
**Table S7** Results of automatic linear modelling (ALM) with model selection on arctangent transformed root damage data as response variable and ‘Water flow’ and ‘DNA’ as categorical predictor from data on the second experiment.
**Table S8** Post hoc results from Duncan test in variation of root damages in *Alnus* roots after application of self‐DNA and nonself‐DNA, and without DNA (control).
**Table S9** Post hoc results from Duncan test in variation of root damages in *Alnus* roots after application of self‐DNA and nonself‐DNA, and without DNA (control) in different water flow conditions.
Please note: Wiley Blackwell are not responsible for the content or functionality of any Supporting Information supplied by the authors. Any queries (other than missing material) should be directed to the *New Phytologist* Central Office.

## Data Availability

Data sharing is not applicable to this article as no datasets were generated or analysed during the current study beyond those reported in the paper.
