# Supplementary material for: Field evidence for litter and self‐DNA inhibitory effects on Alnus glutinosa roots
Source: New Phytol. 2022 Aug 10;236(2):399–412. doi: 10.1111/nph.18391 (PMC9805126; doi:10.1111/nph.18391)
Supplement: Supplementary file 1 — Fig. S1 Selected pictures of the study site in June 2018 showing the formation of leaf litter and wood debris packs, also note the amount of standing litter in the ponds. Fig. S2 Picture of the study site in June 2019 showing a typical pond in the stream with a large Alnus glutinosa tree (right, upper corner). Fig. S3 Heat‐plot of correlation (Pearson’s r) between Alnus glutinosa root damage and litter chemical descriptors in either closed or open experimental systems. Table S1 Physical and chemical parameters of the soil of the study site. Table S2 Results of univariate factorial ANOVA of variation of root damages, and histological sections depending to Alnus plant as random factor and litter type, litter age, water flow if in open/closed systems as fixed factors and interactive effect between the treatments. Table S3 Results of automatic linear modelling (ALM) with model selection on arctangent transformed root damage data as response variable and ‘water flow’ and ‘litter species’ as categorical predictor. Table S4 Post hoc results from Duncan test in variation of root damages in Alnus roots treated with litters of different age (days of decomposition) and type (species) and in different water flow if in open/closed systems. Table S5 Results of automatic linear modelling (ALM) with model selection on arctangent transformed root damage data as response variable and ‘%D’, ‘TEL’, ‘S : R’, and ‘NEL’ as categorical predictor from histological analysis of roots of Alnus glutinosa. Table S6 Results of univariate factorial ANOVA of variation of root damages after application of self‐DNA and nonself‐DNA, depending on the Alnus plant as a random factor and DNA, water flow if in open/closed systems as fixed factors and interactive effect between the treatments. Table S7 Results of automatic linear modelling (ALM) with model selection on arctangent transformed root damage data as response variable and ‘Water flow’ and ‘DNA’ as categorical predictor from data on the second exp [file NPH-236-399-s001.pdf]

Title

Field evidence for litter and self-DNA inhibitory effects on *Alnus glutinosa* roots

Authors:

Giuliano Bonanomi, Maurizio Zotti, Mohamed Idbella, Pasquale Termolino, Veronica De Micco, Stefano Mazzoleni

Article acceptance date: 6 July 2022

SUPPLEMENTARY MATERIALS

Figures S1, S2, S3
Tables S1- S9

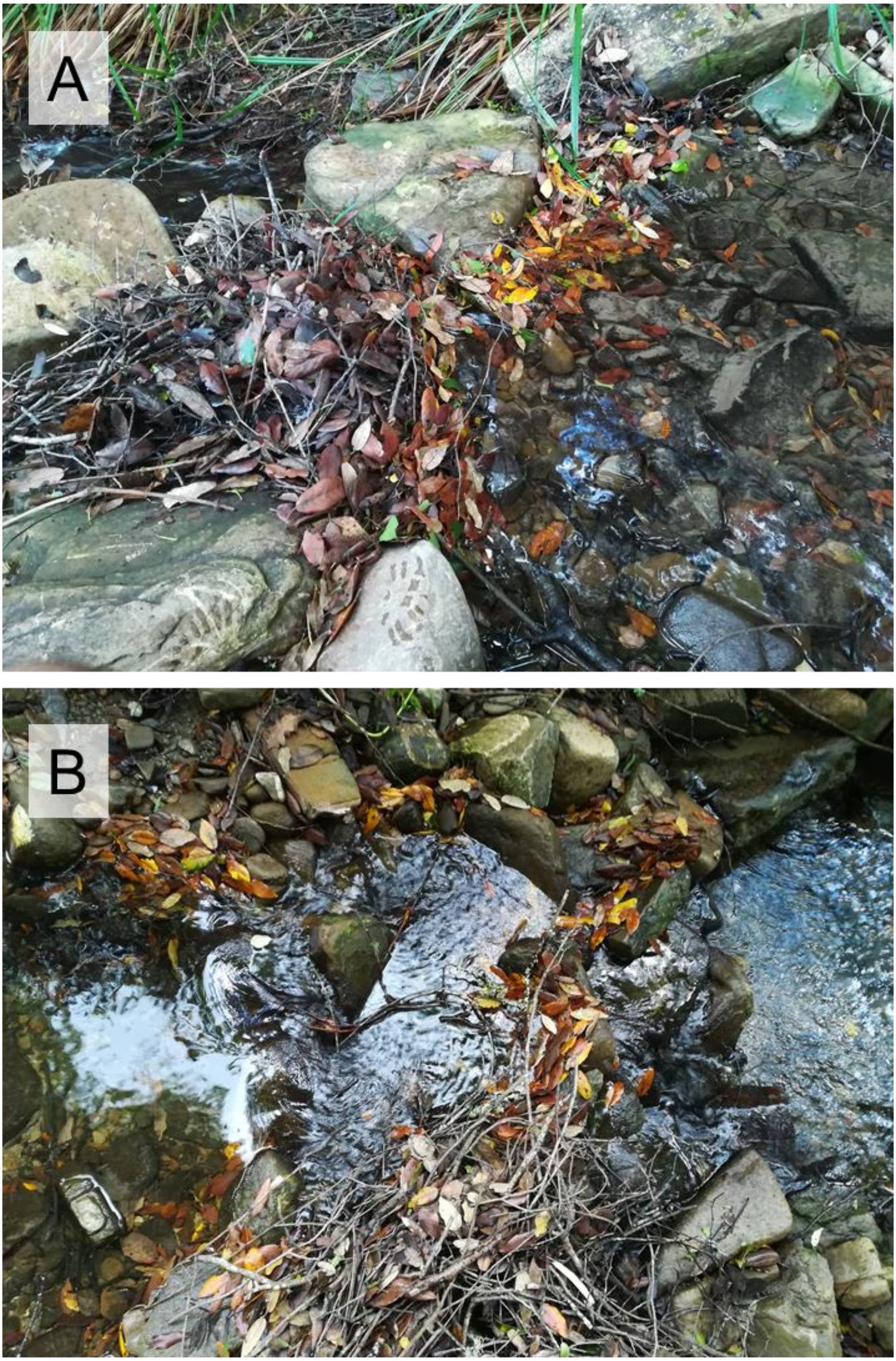

Figure S1. A, B: selected pictures of the study site in June 2018 showing the formation of leaf litter and wood debris packs, also note the amount of standing litter in the ponds. An even higher litter concentration is commonly observed in July and August because of the reduced water flux of the stream.

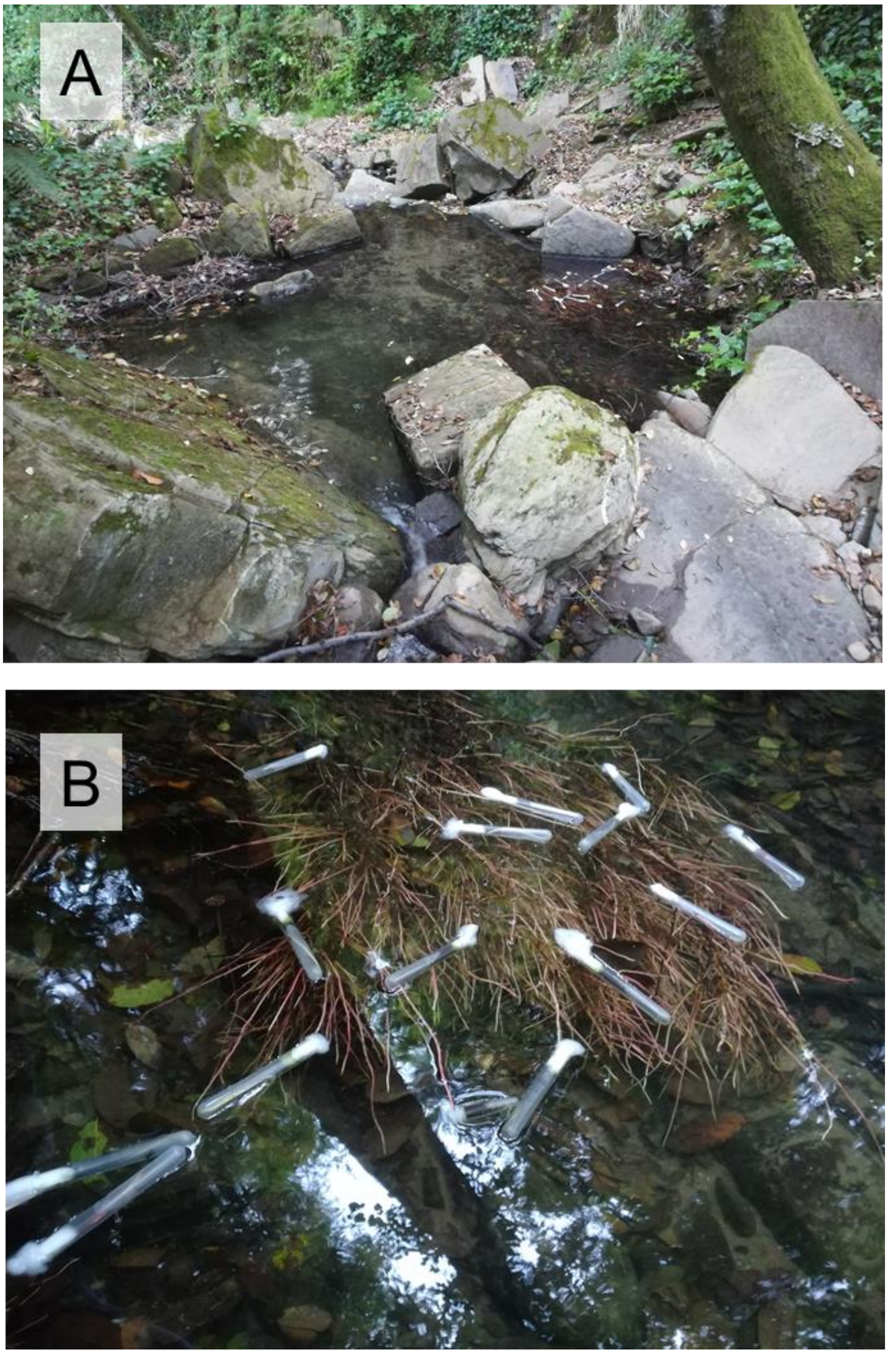

Figure S2. A: picture of the study site in June 2019 showing a typical pond in the stream with a large *Alnus glutinosa* tree (right, upper corner). B: tubes applied to *Alnus glutinosa* tree to challenge the roots with and self- and nonself-DNA.

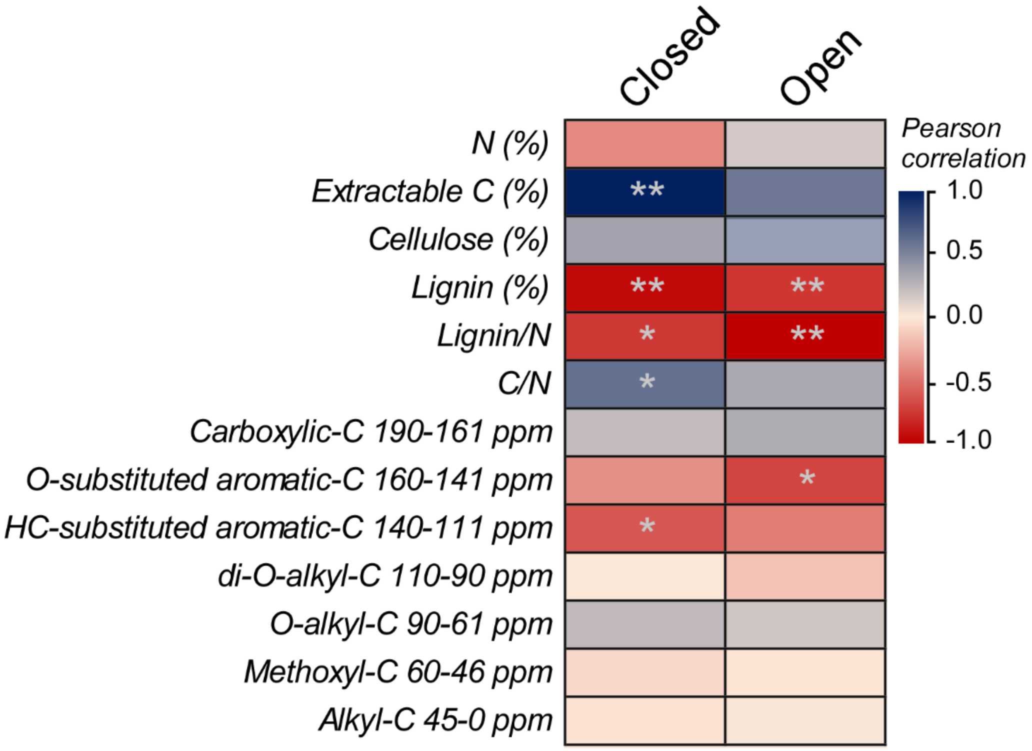

Figure S3. Heat-plot of correlation (Pearson's r) between *Alnus glutinosa* root damage and litter chemical descriptors in either closed or open experimental systems. Asterisks indicate significant correlation values (Pearson's *r*; **, P < 0.001; *, P < 0.05), either negative (red) or positive (blue).

Table S1. Physical and chemical parameters of the soil of the study site. Data are averages ±SD.

**Parameters**	**Riparian forest (Cicerale)**
Sand (g kg^{-1})	622 ± 97
Silt (g kg^{-1})	225 ± 458
Clay (g kg^{-1})	172 ± 32
pH	6.89 ± 0.22
EC (dS m^{-1})	0.10 ± 0.02
Organic C (g kg^{-1})	28.11 ± 3.2
Total N (g kg^{-1})	3.11 ± 0.32
Limestone (g kg^{-1})	2.35 ± 1.32
P_2O_5 (mg kg^{-1})	20.31 ± 2.21
CEC (meq 100 g^{-1})	27.31 ± 5.7
K^+ (meq 100 g^{-1})	2.16 ± 0.53
Mg^{2+} (meq 100 g^{-1})	3.22 ± 0.37
Ca^{2+} (meq 100 g^{-1})	43.38 ± 3.91
Na^+ (meq 100 g^{-1})	0.22 ± 0.72
Cu (mg kg^{-1})	0.98 ± 0.13
Zn (mg kg^{-1})	1.19 ± 0.12
Mn (mg kg^{-1})	6.15 ± 1.15

Table S2. Results of Univariate Factorial ANOVA of variation of root damages, and histological sections depending to Alnus plant as random factor and litter type, litter age, water flow if in open/closed systems as fixed factors and interactive effect between the treatments. In bold significant p-values below 0.05. R: random, F: fixed, n.c.: not computed.

			Root damage		%D		TEL		NEL		S/R	
	Type	Df	F	p	F	P	F	p	F	p	F	p
Alnus plant [1]	R	4	1.656	0.774	n.c.	n.c.	0.666	0.695	n.c.	n.c.	1.242	0.393
Litter type [2]	F	3	50.303	**0.000**	14.940	**0.000**	3.311	0.057	2.534	0.105	1.336	0.308
Litter age [3]	F	1	14.360	**0.018**	1.715	0.255	5.512	0.078	7.445	**0.049**	1.975	0.223
Water flow [4]	F	1	233.291	**0.000**	171.110	**0.000**	64.855	**0.001**	90.326	**0.000**	48.648	**0.002**
Alnus plant*Litter type	R	12	0.927	0.617	0.903	0.609	1.119	0.461	0.717	0.710	n.c.	n.c.
Alnus plant*Litter age	R	4	0.706	0.645	1.143	0.731	1.678	0.340	0.159	0.951	n.c.	n.c.
Litter type *Litter age	F	3	5.143	**0.015**	24.631	**0.000**	5.177	**0.016**	7.355	**0.005**	2.726	0.090
Alnus plant*Water flow	R	4	0.923	0.618	0.124	0.957	0.210	0.918	0.060	0.991	n.c.	n.c.
Litter type *Water flow	F	3	55.835	**0.000**	0.977	0.436	2.875	0.081	1.293	0.322	1.966	0.176
Litter age*Water flow	F	1	5.596	0.071	6.488	0.057	0.211	0.668	5.250	0.082	4.085	0.102
[1]*[2]*[3]	R	12	1.099	0.442	0.834	0.623	1.366	0.312	1.779	0.182	0.468	0.894
[1]*[2]*[4]	R	12	0.702	0.723	1.191	0.397	1.364	0.317	1.568	0.243	0.338	0.847
[1]*[3]*[4]	R	4	1.129	0.393	0.478	0.752	0.900	0.498	2.681	0.093	0.424	0.920
[2]*[3]*[4]	F	3	6.132	**0.012**	9.639	**0.003**	12.035	**0.001**	19.142	**0.000**	0.278	0.840
[1]*[2]*[3]*[4]	R	10	0.432	0.926	3.928	**0.000**	1.226	0.291	1.777	0.082	4.383	**0.000**

Table S3. Results of ALM (automated linear modelling) with model selection on arctangent transformed root damage data as response variable and “water flow” and “litter species” as categorical predictor. Model selection is based on best subgroup selection and AICC (corrected Akaike information criterion). X in table indicates that the effect is reported in the model, - indicate absence of the effect in the model.

	Models			
AICC	5891.172	5910.492	5916.353	5931.971
Water flow**	X	X	-	-
Litter species*	X	-	X	-
Litter age	-	-	-	-

**/*: importance of variable as predictor of root damage

Table S4. *Post-hoc r*esults from Duncan test in variation of root damages in *Alnus* roots treated with litters of different age (days of decomposition) and type (species) and in different water flow if in open/closed systems. In bold significant p-values below 0.05

			0 days decomposition								180 days decomposition							
			Closed				Open				Closed				Open			
			Alnus	*Festuca*	*Populus*	*Edera*	*Alnus*	*Festuca*	*Populus*	*Edera*	*Alnus*	*Festuca*	*Populus*	*Edera*	*Alnus*	*Festuca*	*Populus*	*Edera*
0 days decomposition	Closed	*Alnus*																
		Festuca	0.834															
		Populus	0.991	0.833														
		Edera	0.876	0.943	0.876													
	Open	*Alnus*	0.792	0.942	0.787	0.894												
		Festuca	**0.024**	**0.035**	**0.024**	**0.033**	**0.037**											
		Populus	**0.000**	**0.000**	**0.000**	**0.000**	**0.000**	**0.000**										
		Edera	0.976	0.821	0.982	0.866	0.773	**0.024**	**0.000**									
180 days decomposition	Closed	*Alnus*	0.579	0.708	0.576	0.669	0.743	0.064	**0.000**	0.567								
		Festuca	0.830	0.997	0.829	0.940	0.944	**0.037**	**0.000**	0.819	0.712							
		Populus	0.790	0.943	0.789	0.895	0.995	**0.039**	**0.000**	0.776	0.749	0.945						
		Edera	0.779	0.930	0.773	0.879	0.981	**0.035**	**0.000**	0.759	0.744	0.928	0.979					
	Open	*Alnus*	0.160	0.211	0.160	0.199	0.217	0.339	**0.000**	0.157	0.311	0.217	0.226	0.208				
		Festuca	**0.039**	0.056	**0.040**	0.052	0.057	0.808	**0.000**	**0.039**	0.092	0.058	0.061	0.054	0.437			
		Populus	**0.000**	**0.000**	**0.000**	**0.000**	**0.000**	**0.000**	**1.000**	**0.000**	**0.000**	**0.000**	**0.000**	**0.000**	**0.000**	**0.000**		
		Edera	**0.000**	**0.000**	**0.000**	**0.000**	**0.000**	**0.028**	**0.005**	**0.000**	**0.000**	**0.000**	**0.000**	**0.000**	**0.003**	**0.020**	**0.006**	

Table S5. Results of ALM (automated linear modelling) with model selection on arctangent transformed root damage data as response variable and “%D”, “TEL”, “S/R”, and “NEL” as categorical predictor from histological analysis of roots of Alnus glutinosa. Model selection is based on best subgroup selection and AICC (corrected Akaike information criterion). X in table indicates that the effect is reported in the model, - indicate absence of the effect in the model.

	Models									
AICC	5913.7	5915.9	5918.0	5919.1	5920.1	5920.1	5922.2	5922.3	5.923.2	5924.0
%D***	X	X	X	X	X	X	X	-	-	-
TEL**	X	X	-	X	-	X	-	X	-	X
S/R*	X	X	X	-	X	-	-	X	X	X
NEL		X	X	-	-	X	X			X

***/**/*: importance of variable as predictor of root damage

Table S6. Results of Univariate Factorial ANOVA of variation of root damages after application of self and nonself DNA, depending to Alnus plant as random factor and DNA, water flow if in open/closed systems as fixed factors and interactive effect between the treatments. In bold significant p-values below 0.05. R: random, F: fixed.

	Type	Df	F	p
Alnus plant [1]	R	3	0.1	0.925
DNA [2]	F	2	20.0	**0.002**
Water flow [3]	F	1	8.3	0.064
Alnus plant*DNA	R	6	2.0	0.211
Alnus plant*Water flow	R	3	2.7	0.137
DNA*Water flow	F	2	19.6	**0.002**
[1]*[2]*[3]	R	6	0.6	0.697

Table S7. Results of ALM (automated linear modeling) with model selection on arctangent transformed root damage data as response variable and “Water flow” and “DNA” as categorical predictor from data on the second experiment. Model selection is based on best subgroup selection and AICC (corrected Akaike information criterion). X in table indicates that the effect is reported in the model, - indicate absence of the effect in the model.

	Models			
AICC	133.362	153.647	163.472	172.899
DNA**	X	X	-	-
Water flow*	X	-	X	-

**/*: importance of variable as predictor of root damage

Table S8. *Post-hoc* results from Duncan test in variation of root damages in *Alnus* roots after application of Self- and nonself-DNA and, without DNA (control). In bold significant p-values below 0.05

DNA	Control	Nonself-DNA	Self-DNA
Control			
Nonself-DNA	**0.048**		
Self-DNA	**0.000**	**0.000**	

Table S9. *Post-hoc* results from Duncan test in variation of root damages in *Alnus* roots after application of Self- and nonself-DNA and, without DNA (control) in different water flow conditions. In bold significant p-values below 0.05

DNA	Water flow	Control		Nonself-DNA		Self-DNA	
		Closed	Open	Closed	Open	Closed	Open
Control	Closed						
	Open	0.140					
Nonself-DNA	Closed	**0.000**	**0.010**				
	Open	0.947	0.135	**0.000**			
Self-DNA	Closed	**0.000**	**0.000**	**0.002**	**0.000**		
	Open	**0.002**	0.060	0.377	**0.002**	**0.000**	
